# A Method for Determining Parameters of Mid-Scale Traveling Ionospheric Disturbances

**DOI:** 10.3390/s25175377

**Published:** 2025-09-01

**Authors:** Alexey Andreyev, Vitaliy Kapytin, Artur Yakovets, Yekaterina Chsherbulova

**Affiliations:** Institute of Ionosphere, Almaty 050020, Kazakhstan; alexey.andreyev@ionos.kz (A.A.); chsherbulova@ionos.kz (Y.C.)

**Keywords:** GNSS TEC, TIDs, ionosphere, seasonal variations, magnetically quiet

## Abstract

The growing amount of available experimental data on the Earth’s ionosphere total electron content measurements requires the development of modern data processing instruments. Traveling ionospheric disturbances (TIDs) are one of the ionospheric phenomena that are of great interest. To automate the processing of TEC horizontal distribution maps for studying TIDs, a method for detecting TIDs and their parameters, such as amplitude, speed, and direction of propagation has been developed. It is based on determining the signal-to-noise ratio of TEC variations. The proposed method was used to analyze the TID distribution throughout the United States for more than 40 magnetically quiet days in 2023. The results of the obtained statistical characteristics of the detected TIDs are presented.

## 1. Introduction

In recent years, due to the growth of the density of regional GNSS CORS station networks, it has become possible to map the distribution and dynamics of the total electron content (TEC) of the ionosphere based on the GNSS TEC method with high resolution and at the same time in a large range of latitudes and longitudes [1]. This method allows many researchers from all over the world to confirm the generation of ionospheric disturbances by processes both from the Earth’s surface [2,3] and from space sources. The TEC data showed disturbances from major meteorological phenomena [4,5], orographic disturbances [6], geologic hazards including earthquakes, tsunamis, and volcanic eruptions [7,8,9,10,11,12], rocket launches [13,14], the solar terminator, magnetic storms and solar flares [15].

Basically, these studies consider two-dimensional horizontal TEC maps on a sphere or its segment with a radius corresponding to the height of the maximum of the ionosphere F2 layer (300–350 km), also known as a thin shell approach [16,17]. This approach also has its limitations, which include uneven coverage of the GNSS receiver network, as well as the fact that, in reality, ionospheric disturbances have an inclined structure in the vertical plane [18], i.e., a dependence on the sounding angle [19]. In addition, although the largest electron concentration value is observed at the height of the F2 layer maximum, ionospheric disturbances do not necessarily propagate at this height [20]. Despite this, with a high density of ground-based receivers (currently exceeding 1 receiver per 50 km in some areas of the US and Japan), and a large number of GNSS satellites (currently more than 140 available satellites), this method is widely used and provides a good spatial resolution in the range of tens of kilometers when studying ionospheric disturbances of various scales.

Horizontal dynamic TEC maps are probably the most understandable and visual way of presenting information about ionospheric disturbances. In a number of cases, such as disturbances from earthquakes and rocket launches, it was possible to clearly and intuitively identify the source of disturbances on TEC maps. Horizontal TEC maps alone are understandable and visual but not very suitable for studying TID on long time intervals and for collecting TIDs statistics due to poor automation of processing methods [21]. To obtain TID characteristics such as propagation velocity and direction, the method of constructing range-time or travel-time diagrams is widely used [4,8,22]. Travel-time diagrams are constructed either indicating the distance along some selected direction (e.g., meridional, northward–eastward TIDs are being studied) [16,23] or from a certain geographic point, when studying concentric disturbances (e.g., the ionospheric response to earthquakes or volcanic eruptions) [24] or in some other way, such as disturbances from a moving source [14]. Due to the fact that TIDs usually do not propagate like a plane wave, constructing range-time diagrams along a selected direction has its limitations.

Another method used for automated extraction of TID characteristics is the 3D spectral analysis in lat/lon/time coordinates [25,26]. However, like plotting travel-time diagrams along a fixed direction, 3D spectral analysis does not work well when studying disturbances that do not propagate as a flat wave. The principle of the spectral analysis method is funded on the calculation of the correlation of the studied data using a harmonic function. This method does not work effectively when applied to short time series, signals far from sinusoidal such as single waves or with a variable period [27]. Methods based on taking into account the deviation of the signal value from the background level work better for detecting such disturbances.

To solve the described problems and create a method for automated processing of dynamic TEC maps, we propose a new method based on determining the signal-to-noise ratio of wave disturbance in a local area. The proposed method differs from travel-time diagrams only in its automation. Identification of the coherent wave pattern is not done by eye, but by calculating the signal/noise ratio, and the centers and directions from which the travel-time diagrams are constructed correspond to all map points and all directions.

In this article, we will consider the application of this method using the example of various days during 2023 with low geomagnetic activity. We decided to consider the undisturbed ionosphere for two reasons. Firstly, to obtain background ionospheric disturbances that are created by regular sources, which will later enable us to distinguish disturbances created by sporadic phenomena from their background. And secondly, to ensure the repeatability of obtained TIDs parameters on regular disturbances. It is assumed that the studied TIDs are caused by acoustic-gravity waves (AGW) propagating horizontally.

## 2. Materials and Methods

In this study, data from permanent GNSS receivers (CORS stations, and the Continuously Operating Reference Station, https://geodesy.noaa.gov/CORS/, accessed on 17 July 2025) located in the United States and provided by NOAA (National Oceanic and Atmospheric Administration, USA) were used. All observational RINEX (Receiver Independent Exchange Format) files were downloaded from the website https://geodesy.noaa.gov/corsdata/rinex/ (accessed on 17 July 2025). The NOAA CORS station network was chosen due to its extensive coverage, high and relatively uniform station density, as well as easy access to data.

For this research, we utilized data from all receivers located within the region 20° N–50° N; 60° W–125° W, which includes over 1500 receivers. Data from observational OBS RINEX [https://igs.org/formats-and-standards/, accessed on 17 July 2025] versions 2 and 3 were used. The values of total electron content were calculated for all receivers using the methodology described in [14,19]. Data from L1/L2 for GPS and GLONASS systems and L1/L5 for the GALILEO system were utilized with corresponding frequency coefficients. Thus, for each GNSS receiver, TEC values at up to three dozen ionospheric points were obtained.

Reading RINEX files and calculating total electron content values were performed using the total electron content data collection and processing system that was developed earlier [28]. This system enables the download and reading of RINEX files for a selected region, and to calculate TEC and coordinates of ionospheric points. Differential code biases (DCB) values [29] were calculated based on the difference between the vertical total electron content values calculated from RINEX and the total electron content values taken from GIM IONEX files (https://igs.org/wg/ionosphere/, accessed on 17 July 2025) for the given date, time, and region for each receiver–satellite pair during the total electron content calculation process. In this work, only variations and not absolute total electron content values were investigated, so obtaining precise DCB values was not critical, as it is done in several works [3,4,5,6]. In fact, calculating DCB values and obtaining absolute total electron content values for each GNSS track is necessary for converting inclined TEC values to vertical [30,31]. Therefore, significant deviations of absolute total electron content values from actual values (meaning inaccurate DCB determination during the transformation from STEC to VTEC) lead to distortion of ionospheric wave amplitudes, reflecting the dependence on elevation angle.

The calculated values were overlaid on a regular spatiotemporal grid with resolutions of 1 min in the time and of 0.25 degrees in the latitude and the longitude, covering the area 20°–40° N and 120°–80° W. This work is devoted to medium-scale disturbances. However, the chosen spatial and temporal scales (0.25 degrees and 1 min) and the density of the GNSS CORS network in the USA allow detection of even small-scale disturbances. Total electron content values were calculated for various heights ranging from 0 to 500 km in 20 km increments. Thus, for each height, a three-dimensional array of dimensions 1440/160/160, corresponding to minute of a day/latitude/longitude, was constructed, with each cell containing the averaged total electron content value of all satellite-receiver tracks passing through the corresponding area at that moment. Empty cells were filled by linearly interpolated values from the nearest points in the horizontal plane. Such maps visually demonstrate the process of propagating ionospheric disturbances; however, obtaining statistical characteristics from these maps is challenging and requires further processing.

**Figure 1:**
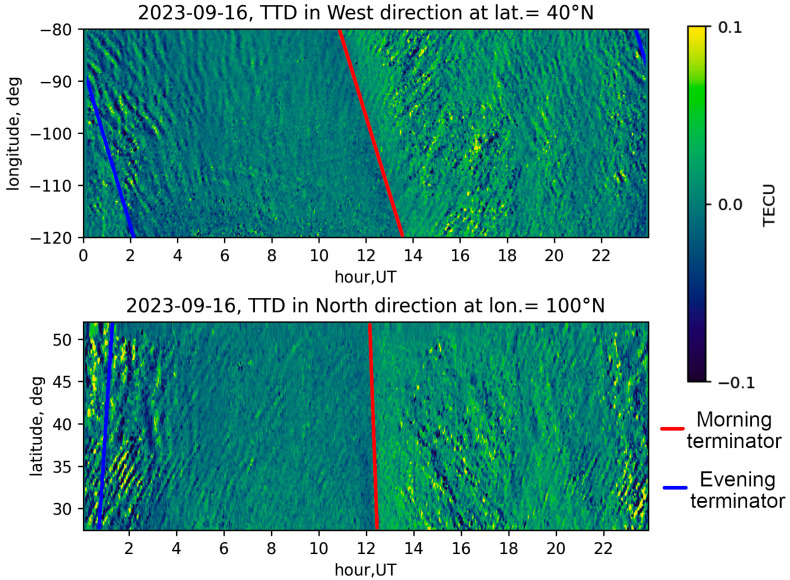
Travel-time diagram of TEC variations on 16 September 2023 drawn along 40° N parallel (**top**) and along 100° W meridian (**bottom**). Morning and evening terminators at the ground level are indicated, respectively, by red and blue lines.

The mid-scale traveling ionospheric disturbances driven by neutral AGW represent regions of increased or decreased electron concentration that propagate in space with a broad front extending several thousand kilometers in horizontal plane [15,32]. Generated by various sources, such as solar terminator, thermal tides, meteorological phenomena, etc., these disturbances could spread simultaneously in the same area of ionosphere in different directions and at varying speeds, leading to a complex interference pattern [25]. Presenting the data in the form of travel-time diagrams resolves this issue and allows for the identification of disturbances with different propagation speeds. Figure 1 shows an example of a travel-time diagram, demonstrating the presence of multiple wave modes. However, since the propagation directions of the disturbances are not constant, the projection of the disturbances on the travel-time diagram will also be changed. To address this issue and automate the process of determining disturbance speeds from travel-time diagrams, the following method is proposed.

**Figure 2:**
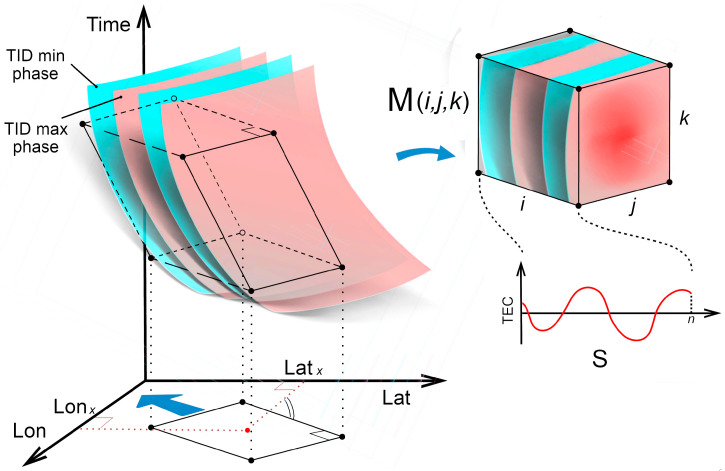
An illustration of the proposed TID detection method.

The propagating TID will appear in space-time as several parallel two-dimensional inclined planes of alternately increased and decreased TEC values, as displayed in Figure 2, corresponding to in-phase regions. The tangent of the angle between the plane and the time axis is equal to the phase velocity of TID, and slope orientation in spatial plane corresponding to propagation direction.

Such disturbances can be detected by measuring the correlation of TEC values on planes oriented in space at different angles corresponding to different directions and velocities of TID propagation. For this purpose, it is possible to consider local areas in space and time, having an extent of several hundred kilometers to several tens of minutes (or several wavelengths to several periods), but defining these areas for each point in space and time. That is, in essence, in the vicinity of each point with coordinates lat_i_/lon_i_ and at each moment of time time_i_, a set of travel-time diagrams are constructed for all directions. By varying the possible propagation velocities of disturbances, the velocities which exhibit important contrast in the wave pattern diagrams are determined. Locality in constructing range-time diagrams in this case solves the problem of determining the speed and direction changing in space, allowing to determine those parameters at each specific point and at each moment of time.

Figure 2 shows an illustration of the described procedure. The inclined parallelepiped with axes «*i*», «*j*» and «*k*» oriented along the azimuth and having a slope corresponding to the propagation velocity of disturbances is transformed into a rectangular array M(*i, j, k*) of shape n × n × n.

When the TID front is oriented along the «*i*» axis, the signal power along the «*i*» axis will exceed the signal power along the «*j*» and «*k*» axes. Thus, we will determine the magnitude of the signal and noise. The signal S will be an array of length n containing averaged values along the «*i*» axis, i.e., parallel planes «*jk*» related to TEC variations. The noise N will be an array of length n containing relative values of TEC dispersion on «*jk*» planes. Accordingly, the signal-to-noise ratio at the geographical point lat_i_/lon_i_, at exact minute of a day time_i_ and for all given variety of speeds and azimuthal directions is defined as the ratio of the average signal power to the average noise power.

$$S_{i}=\frac{1}{n^{2}}\sum_{j,\;k=0\ldots n}M_{ijk}\;$$

$$N_{i}=\frac{1}{n^{2}}\sum_{j,\;k=0\ldots n}(M_{ijk}-{\bar{M_{jk}})}^{2}\;$$

$${SNR}_{\left(lat,\;lon,\;time,\;direction,\;speed\right)}=\frac{\frac{1}{n}\sum_{i=0\ldots n}S_{i}^{2}}{\frac{1}{n}\sum_{i=0\ldots n}N_{i}^{2}}$$

**Figure 3:**
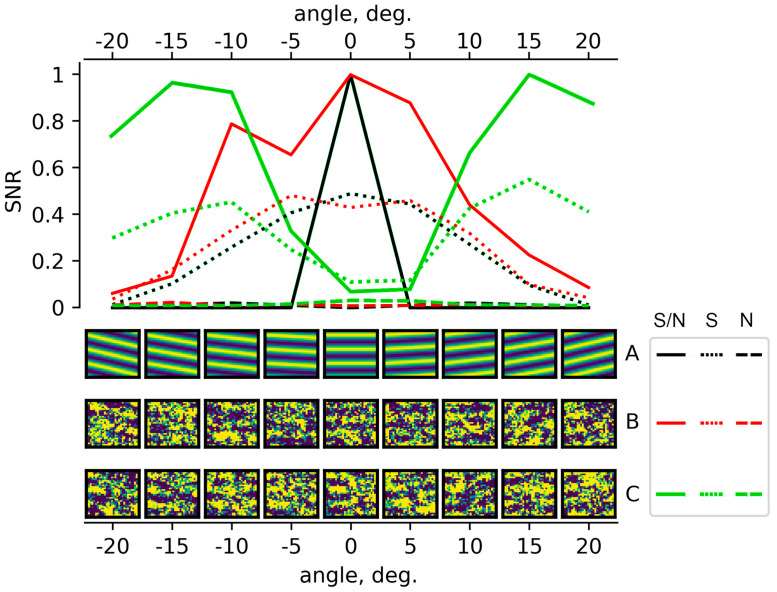
Calculated SNR for synthetic TEC variations. A: sinusoidal wave with amplitude 1.0 traveling at 0 degrees; B: same sinusoidal wave with added random noise; C: two sine waves of same period and amplitude 1.0 traveling in −15° and +15° directions.

In essence, the proposed method of calculating the signal to noise level is probably the simplest and most obvious way to search for wave or any inhomogenous structures in three-dimensional (two spatial coordinates and one time) data. Let us give an example of testing the signal/noise calculation using synthetic harmonical data. Figure 3 shows slices of the M array in the «*ij»* plane (lower panel) for different angles and for three types of synthetic data (A, B, C). The upper panel shows the values of the calculated signal/noise ratio. The synthesized data in Figure 3 are as follows:

A: A sine wave with an amplitude 1.0, propagating in the direction of 0° degrees. This is an example of an ideal TID plane wave. As can be seen, the algorithm clearly determines the traveling direction the signal/noise level.

B: The same sine wave with an amplitude of 1.0, propagating in the direction of 0° degrees, with added random noise, with amplitudes of 2.0. As can be seen, even in this case, the maximum signal/noise value corresponds to the direction of the modeled wave.

C: Superposition of two sine waves with amplitudes of 1.0 and propagating in directions of −15° and +15° degrees plus random noise, with amplitudes of 2.0. In this case, the algorithm also detected two signal level maxima, corresponding to the real ones.

Waves of ionospheric disturbances at each point in space and at each moment in time are found as the positions of the maxima on the plane (speed–direction) of the SNR values. After that the values of coordinates, time, wave speed, direction and amplitude are recorded in a database for further statistical analysis. The amplitude of the TID corresponds to the magnitude of the signal, and not to the magnitude of the SNR, which is used only to detect maxima. Taking into account the noise level it is necessary to increase the maxima accuracy, as shown in Figure 3.

In this study, we focused on detecting TIDs that are not associated with geomagnetic activity, but generated by regular sources: sunrise–sunset events (passage of the solar terminator) and traveling mid-scale atmospheric disturbances. Such TIDs appear on dynamic TEC maps as surface waves on water, and the mechanism of their generation is apparently AGWs generated in the lower atmosphere [2,3,33]. We selected 41 days of 2023 with low geomagnetic activity (Table A1), for which the directions, speeds, coordinates and time of TID observations were determined using the described method.

The following settings were used to determine the TIDs: the local array (array M on Figure 2 and Figure 3) size = 3 × 20 pixels; spatial resolution of the original TEC grid: 0.25° in latitude and longitude; time resolution: 60 s; direction bearing step: 10°; and velocity variation range: 0–1650 km/h.

## 3. Results and Discussion

**Figure 4:**
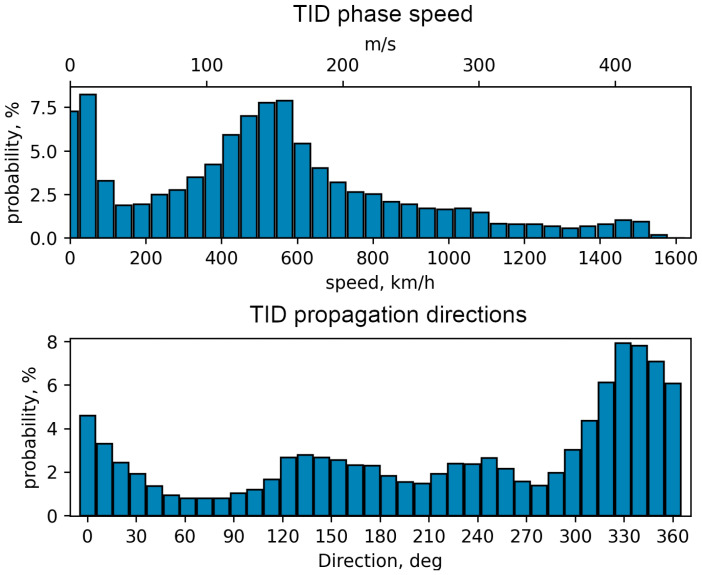
Averaged phase speed and directions (azimuth from north) at all points of map.

Figure 4 shows the distribution of the detected ionospheric disturbances by TID phase speed (upper panel) and TID propagation directions (lower panel). The averaged values for all 41 days are shown, averaged for all coordinate points with a step of 1 degree. As can be seen from the figure, most of the detected TIDs have a phase velocity of about 150 m/s, which is close to values derived in [34,35]. It is worth noting that this velocity changes little during the year, but has a significant variation of more than 50% during each day.

Such a large variations of propagation speeds and directions as seen in Figure 4 are not caused by the inaccuracy of the method, but by a wide range of TID parameters which strongly depends on the geographic coordinates of the ionospheric point and time.

**Figure 5:**
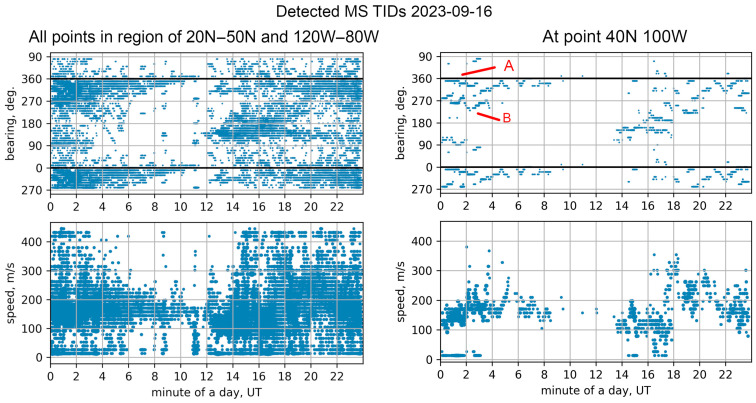
Directions (azimuth from north) and speeds of detected TID, 16 September 2023, at all points of map (**left pane**) and at point 40° N 100° W (**right pane**). The wave groups moving in northern and western directions labeled A and B respectively.

Thus, we will give an example of the dynamics of TID directions and phase velocities for one day: 16 September 2023. Figure 5 shows the total dynamics of TID at 25 points in the region of 20° N–50° N and 120° W–80° W with a step of 5 degrees (left panel), and the dynamics at one point with coordinates 40° N, 100° W (right panel). As can be seen from the total graph, at the beginning of the day the dispersion of direction values is about 90 degrees, and the speed dispersion are up to 100 m/s. However, from the right pane, constructed for one point, it is clear that the method allows tracking the directions and speed of TID with sufficient accuracy. Thus, during the first three hours of the day, according to the Figure 4 right pane, two approximately perpendicular groups of waves are observed, propagating in directions of approximately 270 and 360 degrees, and having a propagation speed varying from 120 to 180 m/s.

Corresponding static TEC maps do not provide a sufficient understanding of the TID dynamics. Therefore, for clarity and ability to compare TID detection results presented in this work with TEC variation maps, we provided animations and a set of plots, which are available at the following link: (http://geomag.ionos.kz/tec_project/tecdata.html, accessed on 10 July 2025). The horizontal TEC map corresponding to this moment in the area of the point 40° N 100° W contains two groups (A and B) of TIDs that converge, moving in the vicinity of this point in the northern and western directions. Those two groups of waves correspond to two tracks in the area of 0–200 min in Figure 5 (i.e., right pane). At the same time, the fact that TIDs do not propagate as a flat front, but often have a concentric or bow shape, leads to a spread of the measured direction values (Figure 5, left pane).

The permissible volume of the article limits us in providing additional examples, but we can state that the described technique allows us to detect the overwhelming majority of TIDs that can be distinguished by the eye when viewing the animation of the TEC dynamics. The method described allows us to determine the directions of TID propagation, their location and time with good accuracy. At the same time, when determining the propagation speed, a significant spread of speed values is observed, exceeding that when determining the speed by the angle of inclination of the wave pattern on the range-time diagrams.

**Figure 6:**
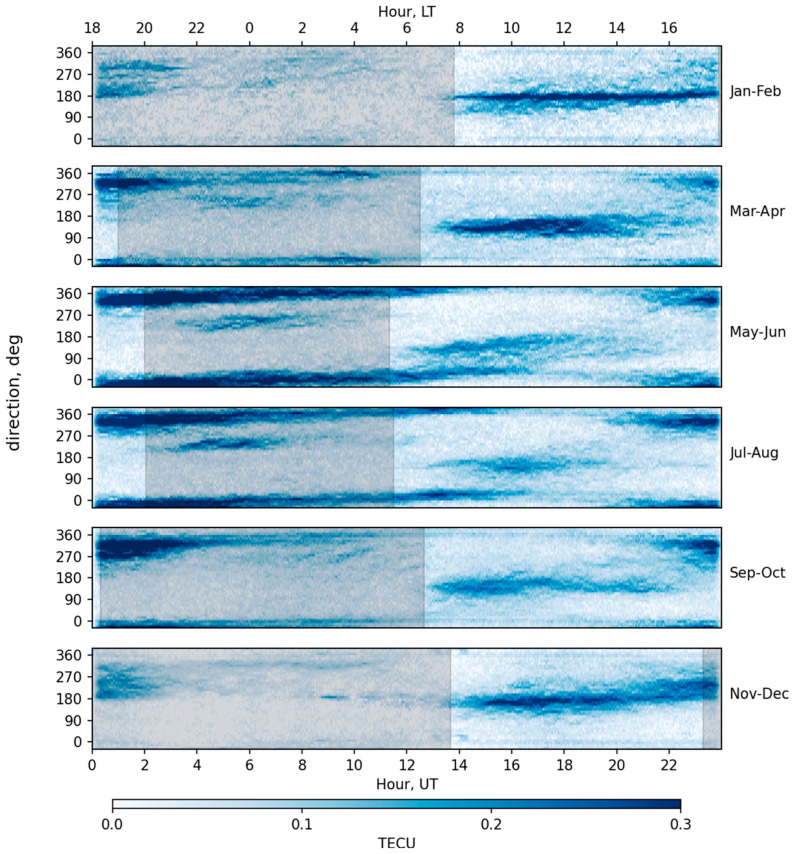
Distribution of TID propagation directions by day and time for different seasons.

Figure 6 shows the distribution of TID amplitudes depending on the propagation direction (azimuth from north) and time of day. The distribution for different seasons of 2023 is given. The distribution is calculated for all points in the rectangle of coordinates 35° N–45° N by 110° W–90° W, as having a high density of GNSS receivers.

As can be seen from the figure, three areas of TIDs are clearly distinguished: a «daytime» and two «nighttime». We would like to note that by highlighting these three areas, we are not classifying TIDs; all of them can be classified as medium-scale TIDs. We are only pointing out that they can be clearly identified by their characteristics—time of occurrence and travel direction. Daytime TIDs that occur immediately after sunrise are observed throughout the year, but in the summer months their intensity and duration are 2–3 times less than in the winter months. Nighttime TIDs propagate mainly in the southern direction in the winter months and in the south-southeast in the summer months. Nighttime TIDs are also observed throughout the year, but in the winter months their intensity is low. In the summer months, nighttime TIDs occur several hours before sunset and continue until sunrise, propagating in the northwest–west direction. It is worth paying special attention to the fairly stable behavior of nighttime TIDs observed from April to August in the period from 21 to 02 h LT and propagating in the southwest direction. Their intensity is low compared to the main mode of nighttime disturbances, but they are clearly detected by this method. The occurrence of time of night medium-scale TIDs are in good agreement with results obtained in [36], and the time of appearance and types of TIDs are consistent with [37].

It is worth mentioning that since the days with very quiet magnetic conditions were used and a large number of days were analyzed, the given TID distribution can be considered as background level of wave disturbances in the ionosphere. In addition to the noted TID types, a large number of other types of disturbances are observed on the dynamic TEC maps, but due to the fact that they are not regular, they do not make a noticeable contribution to the given statistics.

**Figure 7:**
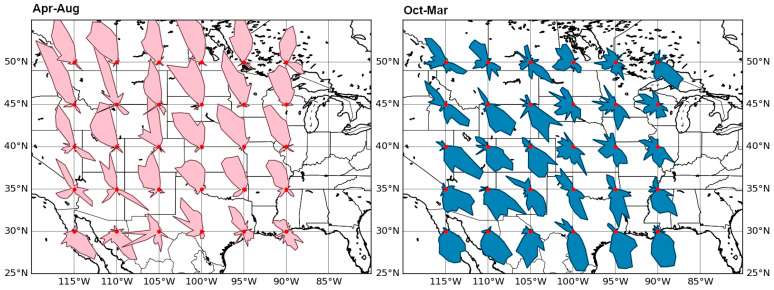
Distribution of year averaged TID propagation directions for different coordinates.

Figure 7 shows the distributions of the predominant directions of the detected TIDs for the summer and winter months separately at different geographical points. The strong latitudinal and longitudinal anisotropy of propagation directions can be seen. Such data on the statistics of the propagation directions and the time of TID occurrence can be used for further comparison with the wind directions in order to establish this type of TID generation mechanism.

## 4. Conclusions

We have described the method developed for automated TID detection, based on determining the signal-to-noise ratio of TEC variations in small vicinities of each specific point in coordinates and time. In its essence, the proposed method of calculating the signal-to-noise level is probably the simplest and most obvious way to search for wave or any inhomogenous structures in three-dimensional data (i.e., two spatial coordinates and one time). We have applied this method to analyze the dynamics of mid-scale TIDs for a significant number of magnetically quiet days in 2023.

The obtained results for medium-scale TID speed and direction distribution are close to the results obtained by other authors. Also, the types of TIDs by seasons of the year (Figure 6), phase velocities, and the time of appearance of medium-scale night TIDs are in good agreement with the results obtained in other works. It is worth recalling that we are talking about mid-latitude (the studied region is 30° N–50° N) disturbances. Moreover, since the TID speeds are determined in the vicinity of each individual point, the speed values obtained by the proposed method should be more accurate than the values determined by the range-time diagrams, which are usually constructed for only one or a few points. So, it is not clear if the TID speed variations showed in Figure 5 are due to the limited sensitivity of the method or whether they are due to real modulation of the apparent TIDs velocities by, presumably, wind currents or horizontal periodical air movements caused by other AGWs. The obtained results on the statistics of TIDs, since they exclude disturbances from magnetic storms, can be called a regular background of ionospheric disturbances created by regular sources such as the solar terminator, atmospheric tides and regular winds.

The disadvantages of the described method include the impossibility of determining the wavelength of ionospheric disturbances. This is due to the fact that the method used is based on the detection of any moving heterogeneity, not only periodic disturbances but also single ones. At the same time, this function can be realized with further development of the method.

In this work, we have applied the described method to real ionospheric TEC data for the first time. In the future, it will be necessary to optimize and compare with the results of other methods and measurement techniques. We believe that after probation this method will provide a large, statistically significant amount of new data on traveling ionospheric disturbances and their generation. The described method can easily be applied to the detection of concentric and bow-shaped ionospheric disturbances generated by point sources, as well as local and short-term wave disturbances.

## Data Availability

The work is based on GNSS RINEX data provided by the NOAA Continuously Operating Reference Station (CORS) Network (NCN), managed by NOAA/National Geodetic Survey via https://geodesy.noaa.gov/CORS/ accessed on 10 July 2025.

## Abbreviations

The following abbreviations are used in this manuscript:

TID	Traveling ionospheric disturbances
TEC	Total electron content
GNSS	Global Navigation Satellite System
CORS	Continuous Operating Reference Station
DCB	Differential Code Biases
AGW	Acoustic gravity waves

## Appendix A

**Table A1:** List of used magnetically quiet days.

No.	Date	Max Ap-Index	Mean Ap-Index
1	9 January 2023	20	11
2	29 January 2023	20	10
3	13 February 2023	20	9
4	24 February 2023	23	11
5	1 March 2023	30	17
6	8 March 2023	30	17
7	17 March 2023	23	13
8	28 March 2023	17	8
9	8 April 2023	20	12
10	16 April 2023	17	9
11	3 May 2023	20	6
12	18 May 2023	7	2
13	26 May 2023	17	13
14	7 June 2023	20	7
15	8 June 2023	17	11
16	27 June 2023	23	20
17	1 July 2023	20	11
18	2 July 2023	17	10
19	4 July 2023	10	8
20	23 July 2023	23	14
21	24 July 2023	23	15
22	15 August 2023	10	6
23	23 August 2023	17	6
24	29 August 2023	20	9
25	4 September 2023	27	15
26	10 September 2023	13	5
27	11 September 2023	20	13
28	16 September 2023	30	12
29	21 September 2023	27	21
30	23 September 2023	27	20
31	3 October 2023	23	16
32	8 October 2023	23	15
33	11 October 2023	10	5
34	23 October 2023	7	2
35	3 November 2023	10	5
36	29 November 2023	17	10
37	7 December 2023	20	11
38	11 December 2023	10	2
39	25 December 2023	13	6
40	26 December 2023	17	12
41	28 December 2023	17	4
